# Effect of the nematophagous fungus *Pochonia chlamydosporia* on soil content of ascarid eggs and infection levels in exposed hens

**DOI:** 10.1186/s13071-018-2898-1

**Published:** 2018-05-29

**Authors:** Sundar Thapa, Stig M. Thamsborg, Rui Wang, Nicolai V. Meyling, Tina S. Dalgaard, Heidi H. Petersen, Helena Mejer

**Affiliations:** 1 Section for Parasitology and Aquatic Pathobiology, Department of Veterinary and Animal Sciences, Faculty of Health and Medical Sciences, University of Copenhagen, Dyrlægevej 100, 1870 Frederiksberg C, Denmark; 2 College of Veterinary Medicine, Inner Mongolia Agricultural University, Hohhot, 010018 People’s Republic of China; 3 Section for Organismal Biology, Department of Plant and Environmental Sciences, Faculty of Science, University of Copenhagen, Thorvaldsensvej 40, 1871 Frederiksberg C, Denmark; 4 Section for Immunology and Microbiology, Department of Animal Science, Aarhus University, Blichers Allé 20, Building P25, 3334, 8830 Tjele, Denmark; 5 Section for Diagnostics and Scientific Advice, National Veterinary Institute, Technical University of Denmark, Kemitorvet, 2800 Kgs. Lyngby, Denmark

**Keywords:** *Ascaridia galli*, *Heterakis gallinarum*, Fungus, Biological control

## Abstract

**Background:**

The nematophagous fungus *Pochonia chlamydosporia* can degrade ascarid (e.g. *Ascaridia galli*) eggs in agar and soil *in vitro*. However, it has not been investigated how this translates to reduced infection levels in naturally exposed chickens. We thus tested the infectivity of soil artificially contaminated with *A. galli* (and a few *Heterakis gallinarum*) eggs and treated with *P. chlamydosporia.* Sterilised and non-sterilised soils were used to examine any influence of natural soil biota.

**Methods:**

Unembryonated eggs were mixed with sterilised (S)/non-sterilised (N) soil, either treated with the fungus (F) or left as untreated controls (C) and incubated (22 °C, 35 days) to allow eggs to embryonate and fungus to grow. Egg number in soil was estimated on days 0 and 35 post-incubation. Hens were exposed to the soil (SC/SF/NC/NF) four times over 12 days by mixing soil into the feed. On day 42 post-first-exposure (p.f.e.), the hens were euthanized and parasites were recovered. Serum *A. galli* IgY level and ascarid eggs per gram of faeces (EPG) were examined on days -1 and 36 (IgY) or 40 p.f.e. (EPG).

**Results:**

Egg recovery in SF soil was substantially lower than in SC soil, but recovery was not significantly different between NF and NC soils. SF hens had a mean worm count of 76 whereas the other groups had means of 355–453. Early mature/mature *A. galli* were recovered from SF hens whereas hens in the other groups harboured mainly immature *A. galli*. *Heterakis gallinarum* counts were low overall, especially in SF. The SF post-exposure IgY response was significantly lower while EPG was significantly higher compared to the other groups.

**Conclusions:**

*Pochonia chlamydosporia* was very effective in reducing ascarid egg numbers in sterilised soil and thus worm burdens in the exposed hens. However, reduced exposure of hens shifted *A. galli* populations toward a higher proportion of mature worms and resulted in a higher faecal egg excretion within the study period. This highlights a fundamental problem in ascarid control: if not all eggs in the farm environment are inactivated, the resulting low level infections may result in higher contamination levels with associated negative long-term consequences.

## Background

*Ascaridia galli* and *Heterakis* spp., collectively known as ascarids, are economically important intestinal nematodes of chickens worldwide. *Ascaridia galli* can impair the health [1–3], productivity [4–7] and welfare of chickens [8]. Moreover, *A. galli* can reduce the vaccine efficacy against Newcastle disease [9, 10] and increase the susceptiblilty of chickens to other infectious diseases such as fowl cholera [11]. On rare occasions, *A. galli* can leave the host’s intestine, migrate up the oviduct and become enclosed inside one of the hen’s eggs, which is of aesthetic concern to the consumers [12, 13]. Compared to *A. galli*, *Heterakis* spp. are less pathogenic, but they can act as a vector for *in ovo* transmission of the protozoan *Histomonas meleagridis* to turkeys and chickens [14]. *Histomonas meleagridis* is pathogenic [15, 16] and re-emerging in layer flocks in many European countries, mainly after the ban of the prophylactic use of chemotherapeutics in the European Union (EU) member countries [17–20].

Recent studies have shown that *A. galli* and *Heterakis* spp. are highly prevalent in European organic laying hen flocks [21–23]. Both nematodes have a simple life-cycle that involves a pre-parasitic development phase (i.e. free-living nematode eggs) in the environment such as litter and soil and a parasitic phase in the chicken’s intestine following ingestion of infective eggs [24, 25]. Ascarid eggs have thick shells [26, 27] and they can survive in the outdoor environment for up to 2–4 years [28, 29], but no effective means of inactivating eggs during or after embryonation in the farm yards and pastures are currently available. At present, control of ascarid infections by farmers therefore solely relies on flock treatment with commercial anthelmintics. Of the anthelmintics, only flubendazole and fenbendazole are available for use in layers in the EU [30, 31]. As hens are rapidly reinfected due to continuous exposure to eggs present in the surroundings and do not appear to acquire protective immunity [32–34], repeated treatments are thus necessary. However, overuse of these drugs may over time enhance the risk of selecting for anthelmintic resistance.

To be able to also combat the parasites in the environment, there is an increasing interest in using naturally occurring soil microfungi as is done to control agricultural nematode pests [35]. An isolate of *Pochonia chlamydosporia* (syn. *Verticillium chlamydosporium*) (Ascomycota: Hypocreales), a microfungus of global occurrence [36–38], that can mechanically and enzymatically degrade the egg shell components (protein and chitin) has already been developed as a biocontrol agent against plant-parasitic nematode eggs [39]. The same isolate [40] and two other isolates [41] of *P. chlamydosporia* have subsequently been shown to kill ~70% and > 80%, respectively, of *A. galli* eggs in laboratory agar assays. However, the effect in a soil based assay was relatively lower (~45%) [42]. In the latter study, the fungal effectiveness was evaluated based on eggs recovered from soil before and after the fungal treatment. However, it is unknown if the recovered eggs judged visually as viable are indeed infective and whether the reduced contamination in the soil assay translates to lower worm burdens in chickens exposed to the fungus-treated soil. In addition, literature indicates that population composition of *A. galli* in chickens can be dose-dependent as shown by a reduced number of inhibited larvae in the host’s intestine and a shorter prepatent period in lightly infected compared to heavily infected chickens [43, 44]. It is thus important to use an *in vivo* infection model to assess how changes in exposure level as a result of fungal treatment of soil may modulate worm population dynamics within the host as this may in turn alter on-farm transmission dynamics.

The overall aim of this study was to evaluate the *in vivo* infectivity of soil experimentally contaminated with ascarid eggs and treated with *P. chlamydosporia.* Both sterilised and non-sterilised soils were used to include any effect inherent to the natural soil biota and thus to evaluate the potential of *P. chlamydosporia* as an on-farm biocontrol agent.

## Methods

### Experimental design

Unembryonated ascarid eggs were added to Petri dishes with sterilised (S) or non-sterilised (N) soil, half of which were treated with spores of the fungus *P. chlamydosporia* (F) while the other half were untreated (C). The Petri dishes were incubated at 22 °C for 35 days to allow the fungus to grow and the eggs to reach infectivity. After incubation, three subgroups of 10 hens per soil treatment were exposed in-feed four times to the soil from one of the four treatments (SC, SF, NF and NC). The hens were euthanized on day 42 post-first-exposure (p.f.e.) and examined for *A. galli* and *Heterakis* spp. To estimate the number of eggs in the Petri dishes, recovery of eggs was tested before [i.e. day 0 post-incubation (p.i.)] and after incubation (day 35 p.i.) for each of the four soil treatments.

### Origin and isolation of ascarid eggs

Before collecting faeces, the infection status of *A. galli* and *Heterakis* spp. in a Danish organic layer farm with a flock size of 3000 hens was examined through necropsy of 18 randomly selected hens. The prevalence was 89% for *A. galli* and 100% for *Heterakis* spp., and mean ± S.E. worm burdens were 40 ± 9 and 80 ± 21 worms, respectively. Ascarid eggs (*A. galli* and *Heterakis* spp.) were isolated from fresh hen faeces collected from the ground using only the top part of the faeces as described by Thapa et al. [29]. The eggs were stored in sterile demineralised water at 5 °C for 6 days. Before use, a subsample of eggs was embryonated in 0.1 N H_2_SO_4_ at 25 °C for 15 days to assess percentage embryonation (i.e. ability to develop larva) of the egg batch [42] which was 95 ± 1% (mean ± SE).

### Preparation of fungal inoculum

Parboiled rice initially soaked for 1 h in demineralised water and autoclaved (121 °C, 15 min) inside a polypropylene bag (Labsolute®, 300 g rice per bag) was inoculated with 10 ml *P. chlamydosporia* Biotype 10 spore suspension [5.5 × 10^7^ conidia and 1.6 × 10^4^ chlamydospores in 0.05% Triton® X-100 (Merck KGaA, Darmstadt, Germany) harvested from 5-week-old culture in Sabouraud’s dextrose agar]. After incubating the rice at 25 °C for 25 days in darkness, 30 ml 0.05% Triton® X-100 was added to each 10 g of rice granules in centrifuge tubes and shaken gently to separate spores from the rice. The mixture was filtered through a 900 μm sieve to remove rice particles and centrifuged (1831× *g*, 3 min) three times after re-suspension in 0.05% Triton® X-100. Spore concentration was adjusted to 4 × 10^8^ conidia and 1.5 × 10^5^ chlamydospores per ml suspension. Spore germination was determined [42] to be 96% and 91% for the conidia and chlamydsopores, respectively.

### Preparation of soil

In April 2016, 15 kg sandy loam soil (pH 6.8) was collected from a Danish experimental plot that was established in 2002 and treated anually (2003–2015) with source separated organic household waste compost (CH) and sown with spring cereals [45, 46]. After removing plant material and stones, the soil was sieved (3 mm) and thoroughly homogenised. Three kilograms of soil was sterilised by autoclaving (121 °C, 30 min) inside a polypropylene bag (Labsolute®, 200 g soil per bag, treatment S) while another 3 kg soil was kept without autoclaving (treatment N).

### Fungal treatment of soil and eggs

For both soils (S, N), 44 replicate Petri dishes (14.5 × 2 cm) each containing 46 g soil and 2 ml ascarid egg suspension with approximately 8000 ± 260 eggs (mean ± SE) in sterile water were prepared. Both soil types were randomised into control (C) and fungus treatment (F). To all SF and NF dishes, 2 ml fungal suspension containing approximately 8 × 10^8^ conidia and 3 × 10^5^ chlamydospores of *P. chlamydosporia* in 0.05% Triton® X-100 was added whereas all SC and NC dishes received 2 ml 0.05% Triton® X-100 without fungus. The SC and SF dishes received an additional 635 μl of sterile water to balance the total moisture level between the S and N soils. The dishes were sealed with Parafilm ‘M’® and the initial (i.e. pre-incubation) soil moisture level per dish (23–24% of the total soil weight) was estimated (105 °C, 24 h). Five random dishes per treatment were used to estimate day 0 p.i. egg recovery, while the remaining dishes were incubated at 22 °C for 35 days in darkness. The weight of each incubated dish was recorded at days 0 and 35 p.i. to determine soil moisture loss (%). On day 35 p.i., the dishes were opened and 4 ml sterile water was added to each dish, re-sealed with parafilm and stored at 10 °C for up to 18 days. On day 7 post-storage at 10 °C (i.e. after incubation was terminated), egg recovery (i.e. exposure level) was estimated in five random dishes per treatment (see section on recovery of eggs from soil). Soil from one random dish was selected and administered in the feed to a corresponding subgroup of hens (see section on animal exposure to parasites). This exposure was repeated on days 11, 15 and 19 after incubation was terminated.

### Recovery of eggs from soil

Fifty millilitres of 0.5 M NaOH was added to each of the 40 dishes (*n* = 5 per treatment on day 0 and 35 p.i.) that was then stored at 5 °C for 16 h. The soil was washed through 212 and 20 μm sieves and the material on the latter was divided into four 50 ml tubes, centrifuged at 253× *g* for 7 min and the eggs were recovered as described by Thapa et al. [29]. For each dish, the egg quantity and development stage (unembryonated, pre-larvated, larvated or degenerated) was examined in a 20% subsample at 100× magnification [29].

### Experimental animals and housing

One hundred thirty pullets (ISA Warren, 18-weeks-old), raised indoors without previous anthelmintic treatment, were obtained from a commercial breeder. On arrival (day -15 p.f.e.), 10 randomly selected pullets were euthanized and examined for ascarid infections of the breeder farm-origin (see section on recovery of worms). All necropsied pullets were found positive for tissue phase *A. galli* larvae (~0.5 mm long) with an overall mean ± SE worm burden of 194 ± 97 *A. galli*, while only two birds harboured luminal *Heterakis* spp. giving an overall burden of 1 ± 1 worm per hen. The remaining pullets (*n* = 120), after random allocation into 12 indoor pens (*c.*2.8 m^2^, 10 pullets per pen), were therefore treated with flubendazole (Verminator®, 1.43 mg flubendazole per kg live weight daily) in the feed from days -13 to -6 p.f.e. The individual body weight of all birds was measured on days -1 and 36 p.f.e. The birds were given pelleted feed (17.5% crude protein, 4.5% crude fat) in two meals (110 g feed per bird per day) and water *ad libitum*. Crushed oyster shells were offered daily. Wood-chips and straw were used as bedding material. Pens were enriched with a perch and nests, and cleaned thoroughly once weekly.

### Animal exposure to parasites

The 12 pens were allocated to the four treatment groups (SC, SF, NC and NF) in triplets (i.e. three subgroups per treatment group). The hens were exposed to ascarid contaminated soil on days 0, 4, 8 and 12 p.f.e. to mimic a moderate trickle infection. On each exposure, entire soil from one Petri dish was transferred to a 500 ml container with 150 g feed of the morning meal and 50 ml tapwater, mixed thoroughly and spread in a tray (58 × 21 × 3 cm) in each pen. The feed was eaten within 10–15 min and the remainder of the meal was then given in the same tray.

### Recovery of worms

The hens were euthanized by stunning and cervical dislocation on day 42 p.f.e. The *A. galli* worms in the small intestinal lumen were isolated using an agar-gel method [47] and collected using a 20 μm sieve. The tissue phase larvae of *A. galli* (day -15 and 42 p.f.e.) and *Heterakis* spp. (day -15 p.f.e.) were isolated from the intestinal/caecal tissue by pepsin (1:3000 IU)-HCl (30%) digestion [47] and collected on a 20 μm sieve. To recover luminal *Heterakis* spp. (day -15 and day 42 p.f.e.), the caeca were opened and stored in tap water at 5 °C. After 48 h, the caeca and contents were washed on a 20 μm sieve. All worm samples were stored in 70% ethanol and examined using a dissection microscope (30–40× magnification). All *A. galli* worms were categorised as < 0.5, 0.5–1.5, 1.5–3.0, 3.0–5.0 or 5.0–8.0 cm, whereas *Heterakis* spp. were categorised as < 0.5 or ≥ 0.5 cm. Moreover, *Heterakis* species were determined based on the length of the spicules [48, 49] of 50 randomly selected male worms (1 worm per hen and representing all experimental groups) after exposing each worm to a drop of 10% lactic acid in water (weight/weight).

### Faecal egg counts

Individual faecal samples from all birds were collected on days -1 and 40 p.f.e. Ascarid eggs per gram faeces (EPG) was determined by a concentration McMaster technique (minimum detection limit: 20 EPG) using a flotation fluid of 500 g glucose monohydrate per litre of saturated NaCl solution (specific gravity: 1.27) [50].

### *Ascaridia galli* antibody (IgY) levels

To determine the systemic antibody response as an indirect assessment of parasite exposure, individual blood samples from all birds were collected on days -1 and 36 p.f.e. from a wing vein. Serum was separated by centrifugation at 1000× *g* for 15 min and stored at -20 °C. The *A. galli* IgY level was determined by ELISA according to Norup et al. [51] using crude adult *A. galli* somatic antigens and one replicate serum sample per animal per sampling day. A dilution series of a highly positive serum was used as standard and the highest concentration was set at the relative value 2.

### Statistical analyses

All statistical analyses were performed using SAS 9.4 (Cary, NC, USA). The main and interaction effects of soil sterility (S, N), fungal treatment (C, F) and incubation time (days 0, 35 p.i.) on egg recovery from soil were analysed using a generalised linear model fitted with negative binomial distribution of errors (NBD) (procedure GENMOD). Soil moisture loss during incubation was analysed with a linear model (procedure GLM) with percent moisture loss as the outcome and soil sterility (S, N), fungal treatment (C, F) and their interaction as predictors. Body weight at day 0 p.f.e. and weight gain (days 0 to 36 p.f.e.) in relation to soil sterility (S, N) and fungal treatment (C, F) were analysed separately with a linear-mixed model (procedure MIXED) with subgroup (i.e. pen) as a random effect. Worm burden (total, *A. galli*, *H. gallinarum*), proportion (%) of *A. galli* in the intestinal tissue, proportion (%) of *A. galli* in each length category (< 0.5, 0.5–1.5, 1.5–3.0, 3.0–5.0, 5.0–8.0 cm) and at day 40 p.f.e. EPG was analysed with a generalised linear mixed model (procedure GLIMMIX, NBD) that included soil sterility (S, N), fungal treatment (C, F) and their interaction as fixed effects and subgroup as a random effect. The log-transformed IgY titre was analysed with a linear-mixed model (procedure MIXED) with soil sterility (S, N), fungal treatment (C, F), sampling time (days -1, 36 p.f.e.) and their interaction as fixed effects, subgroup as a random effect and individual bird as a repeated measurement. At group level, the linear relationships between worm burden (total ascarid or *A. galli*) and the IgY titre difference between pre- and post-exposures were examined using a Spearman method (procedure CORR). The goodness of fit of each GENMOD and GLIMMIX model was assessed with the ratio of Pearson’s *χ*^2^ and corresponding degress of freedom. The normality of residuals of each GLM and MIXED model was examined by a q-q plot and a histogram, and homogeneity of residual variance assessed by residual plots. For each model, the *post-hoc* significant differences were determined with the differences of least squares means (Tukey-Kramer’s adjustment for multiple comparisons, *P* < 0.05).

## Results

### Recovery of eggs from soil

**Fig. 1:**
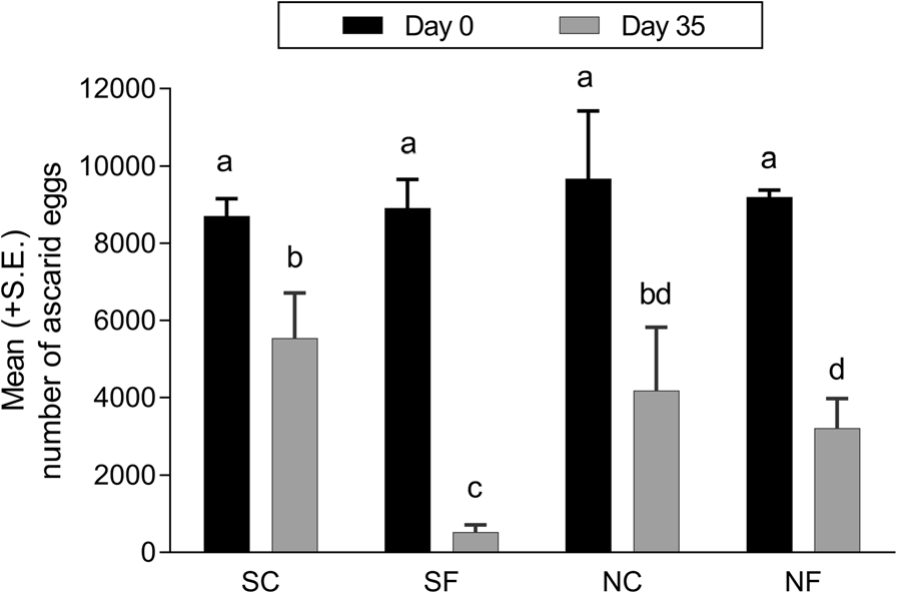
Mean (+ SE) number of ascarid eggs recovered from soil on days 0 and 35 post-incubation at 22 °C. Approximately 8000 unembryonated eggs were added to soil given four different treatments (*n* = 5) (*Abbreviations*: SC, sterilised control; SF, sterilised with the fungus *Pochonia chlamydosporia* Biotype 10; NC, non-sterilised control; NF, non-sterilised with fungus). Different letters above the bars indicate significant differences (*P* < 0.05, Tukey-Kramer’s adjustment for multiple comparisons)

On day 0 p.i., the mean number of eggs recovered from the SC, SF, NC and NF soils were 8702–9673 with no significant differences between the treatments (*P* > 0.9950 in all cases) (Fig. 1). Irrespective of treatment, > 97% of the recovered eggs were unembryonated. On day 35 p.i., the mean egg number in the SC, SF, NC and NF soils was 5535 (36% reduction), 521 (94% reduction), 4176 (57% reduction) and 3201 eggs (65% reduction), respectively (Fig. 1). In the sterilised soil, the fungal treatment resulted in a significant reduction in egg recovery when compared to the control (*P* < 0.0001). In contrast, there was no such difference in the non-sterilised soil (*P* = 0.5480). This meant that there was a strong significant (*χ*^2^ = 70.72, *df* = 4, *P* < 0.0001) interaction between soil sterility, fungal treatment and incubation time on egg recovery. Regardless of treatment, ~94% of the recovered eggs at day 35 p.i. contained a slender larva that resembled the infective stage. The mean ± S.E. moisture loss in the sterilised soil (28 ± 1.8%) was slightly but significantly higher than in the non-sterilised soil (21 ± 1.4%) (*F*_(1, 64)_ = 8.53, *P* = 0.0048).

### Clinical observations and performance

On day 0 p.f.e., the overall mean live weight of hens in the four groups was 1.53–1.60 kg with no significant effect of soil sterility (*F*_(1, 106)_ = 2.29, *P* = 0.1330), fungal treatment (*F*_(1, 106)_ = 1.91, *P* = 0.1701) and their interaction (*F*_(1, 106)_ = 0.82, *P* = 0.3684). By day 36 p.f.e., the overall mean weight gain of hens in the four groups was -76 to 90 g, but there was no significant effect of soil sterility (*F*_(1, 106)_ = 3.39, *P* = 0.0686), fungal treatment (*F*_(1, 106)_ = 0.04, *P* = 0.8367) and their interaction (*F*_(1, 106)_ = 2.72, *P* = 0.1022). Most hens started laying eggs from days 3–7 p.f.e. During the study, the hens showed no overt signs of illness but two hens from one of the three NC subgroups died, possibly due to cannibalism.

### Worm burdens

**Fig. 2:**
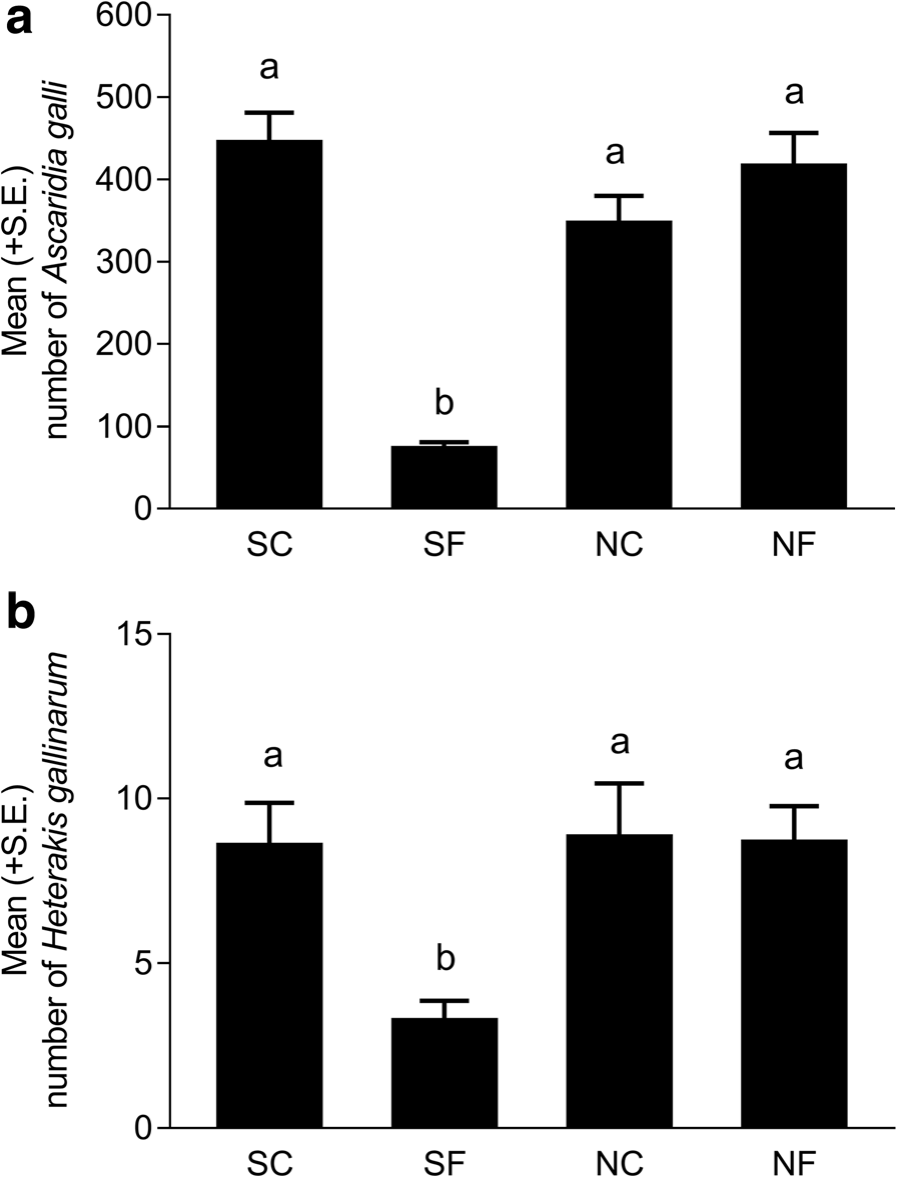
Mean (+ SE) total worm burdens of *Ascaridia galli* (**a**) and *Heterakis* spp. (**b**) recovered from four groups of hens 42 days after the first (of the total four) in-feed exposures to ascarid eggs embryonated in sterilised control soil (SC), sterilised soil with the fungus *Pochonia chlamydosporia* Biotype 10 (SF), non-sterilised control soil (NC) or non-sterilised soil with *P. chlamydosporia* (NF). Each bar represents the mean of 28–30 hens allocated to three replicate subgroups of 8 (one NC subgroup) to 10 hens. Different letters above the bars indicate significant differences (*P* < 0.05, Tukey-Kramer’s adjustment for multiple comparisons)

The overall mean worm burdens of *A. galli* and *H. gallinarum* in hens in the four groups are shown in Fig. 2a and b, respectively. All 118 hens were *A. galli* positive, while 115 birds were *H. gallinarum* positive. The left and right spicules of *H. gallinarum* males had a mean ± SE length of 2086 ± 26 μm (range: 1554–2417 μm) and 723 ± 8 μm (range: 402–850 μm), respectively. The interaction between the soil sterility and fungal treatment strongly influenced the total ascarid worm burden (*F*_(1, 106 )_= 100.38, *P* < 0.0001) and the individual worm burdens of both *A. galli* (*F*_(1, 106)_ = 96.85, *P* < 0.0001) and *H. gallinarum* (*F*_(1, 106)_ *=* 10.07, *P* = 0.0020). Group SF hens thus had significantly lower worm burdens of both *A. galli* (*P* < 0.0001 in all cases) and *H. gallinarum* (*P* ≤ 0.0001 in all cases) compared to the three other groups that all had comparable *A. galli* (*P* > 0.3120 in all cases) and *H. gallinarum* worm burdens (*P* > 0.9989 in all cases) (Fig. 2). *Heterakis gallinarum* represented 6% of the total ascarids for SF hens and 2–3% for the three other groups. With reference to the estimated cumulative egg dose of 2214 (SC), 208 (SF), 1670 (NC) and 1281 eggs (NF) that each hen was theoretically exposed to on four exposures, the overall establishments of total ascarid were 20% (SC), 36% (SF), 21% (NC) and 33% (NF).

### Parasite population composition

**Fig. 3:**
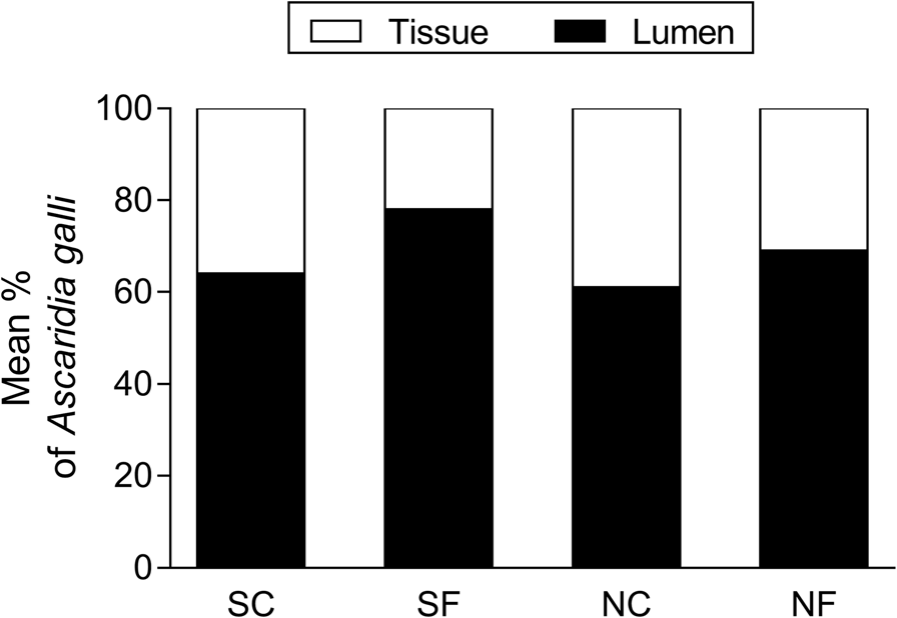
Mean proportion (%) of luminal phase and tissue phase *Ascaridia galli* recovered from four groups of hens 42 days after the first (of the total four) in-feed exposures to ascarid eggs embryonated in sterilised control soil (SC), sterilised soil with the fungus *Pochonia chlamydosporia* Biotype 10 (SF), non-sterilised control soil (NC) or non-sterilised soil with *P. chlamydosporia* (NF). Each bar represents the mean of 28–30 hens allocated to three replicate subgroups of 8 (one NC subgroup) to 10 hens

The overall mean proportion of *A. galli* recovered from the intestinal lumen and intestinal wall is shown in Fig. 3. In general, *A. galli* were more prevalent in the intestinal lumen (61–78%) than in the intestinal tissue (22–39%). Fungal treatment had a significant effect on the relative distribution of tissue phase and luminal phase *A. galli* (*F*_(1, 106)_ = 9.85, *P* = 0.0022). This resulted in a significantly higher proportion (37 ± 2%, mean ± S.E.) of tissue phase *A. galli* in hens not exposed to fungal treatments compared to the hens exposed to fungal treatments (27 ± 2%). There were no significant effects of soil sterility (*F*_(1, 106)_ = 3.05, *P* = 0.0660) as well as the interaction between fungal treatment and soil sterility (*F*_(1, 106)_ = 0.50, *P* = 0.4815) on the *A. galli* distribution between intestinal lumen and tissue.

**Fig. 4:**
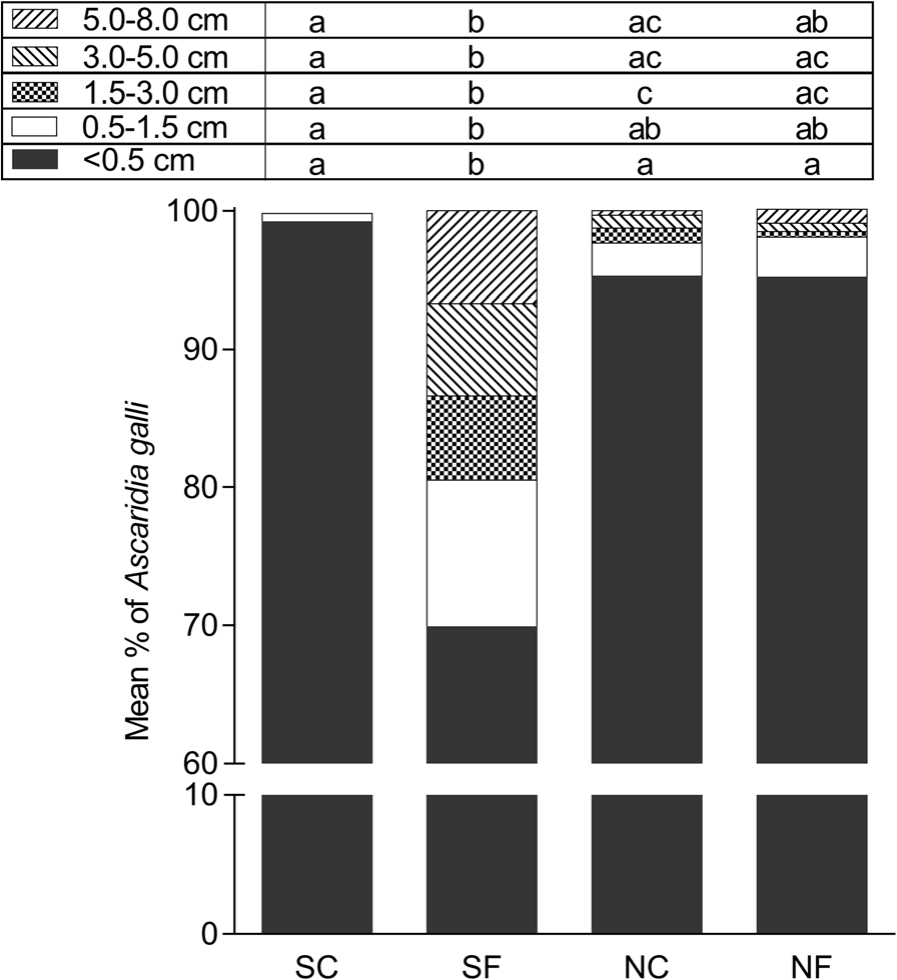
Mean proportion (%) of *Ascaridia galli* of different sizes recovered from four groups of hens 42 days after the first (of the total four) in-feed exposures to ascarid eggs embryonated in sterilised control soil (SC), sterilised soil with the fungus *Pochonia chlamydosporia* Biotype 10 (SF), non-sterilised control soil (NC) or non-sterilised soil with *P. chlamydosporia* (NF). Each bar represents the mean of 28–30 hens allocated to three replicate subgroups of 8 (one NC subgroup) to 10 hens. Different letters above the bars indicate significant differences (*P* < 0.05, Tukey-Kramer’s adjustment for multiple comparisons) within each length category

The proportion of *A. galli* (of the total *A. galli* worm burden) within each length category was significantly related to the interaction between soil sterility and fungal treatment (< 0.5 cm: *F*_(1, 106)_ = 29.48, *P* < 0.0001; 0.5–1.5 cm: *F*_(1, 106)_ = 9.89, *P* = 0.0022; 1.5–3.0 cm: *F*_(1, 106)_ = 29.98, *P* < 0.0001; 3.0–5.0 cm: *F*_(1, 106)_ = 16.58, *P* < 0.0001; 5.0–8.0 cm: *F*_(1, 106)_ = 7.24, *P* = 0.0083) (Fig. 4). Compared to the groups SC, NC and NF hens, the group SF hens hosted a significantly lower proportion of *A. galli* < 0.5 cm (*P* < 0.0001 in all cases) and significantly higher proportions of the three largest length categories (*P* < 0.0225 in all cases, except *P* = 0.0508 for SF *vs* NF in the category 5.0–8.0 cm). The SC, NC and NF hens hosted nearly equal proportions of all five *A. galli* length categories (*P* > 0.0625 in all cases, except *P* = 0.0076 for SC *vs* NC in the category 1.5–3.0 cm). Irrespective of group, all tissue phase *A. galli* larvae were < 0.5 cm (~0.5 mm). In groups SC, NC and NF hens, the luminal *A. galli* worms within the catergory < 0.5 cm (i.e. 5 mm) were approximately 0.5–1.0 mm whereas those in group SF hens ranged ~0.5–4.9 mm.

For *H. gallinarum*, the highest mean ± S.E. proportion of worms > 0.5 cm was hosted by the group SF hens (52 ± 9%) followed by NC (38 ± 6%), NF (31 ± 5%) and SC hens (25 ± 6%). However, the effect of soil sterility, fungal treatment and their inferactions on *H. gallinarum* population composition was not possible to analyse using the same statistical model that was used for *A. galli* because many hens had only < 0.5 or ≥ 0.5 cm *H. gallinarum*.

### Faecal egg counts

**Fig. 5:**
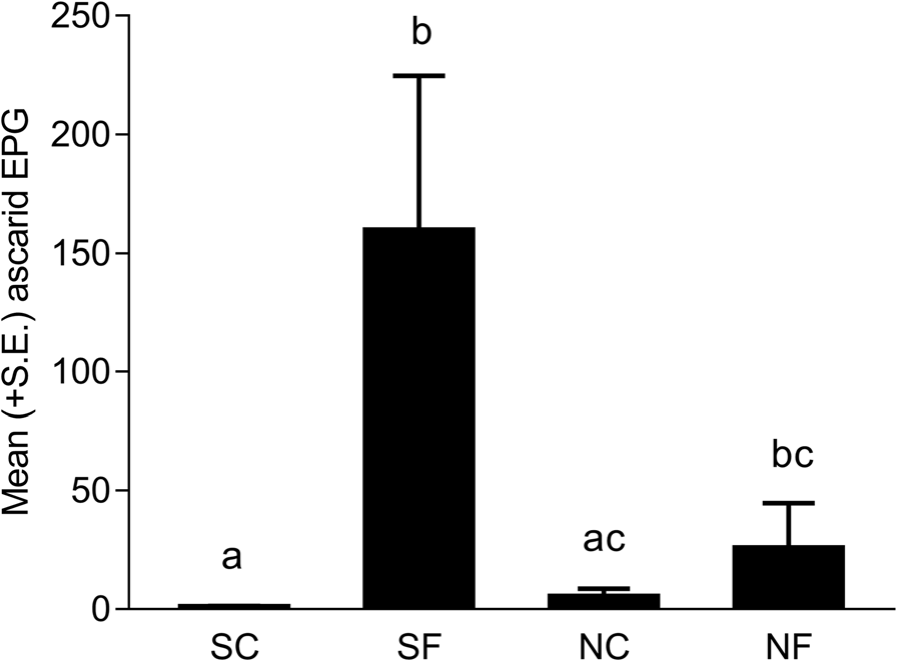
Mean (+ SE) number of ascarid eggs per gram of faeces (EPG) of four groups of hens 40 days after the first (of the total four) in-feed exposures to ascarid eggs embryonated in sterilised control soil (SC), sterilised soil with the fungus *Pochonia chlamydosporia* Biotype 10 (SF), non-sterilised control soil (NC) or non-sterilised soil with *P. chlamydosporia* (NF). Each bar represents the mean of 28–30 hens allocated to three replicate subgroups of 8 (one NC subgroup) to 10 hens. Different letters above the bars indicate significant differences (*P* < 0.05, Tukey-Kramer’s adjustment for multiple comparisons)

On day -1 p.f.e., all hens were negative for ascarid eggs. On day 40 p.f.e., 3, 57, 7 and 17% hens of groups SC, SF, NC and NF, respectively, had positive EPG. There was a significant interaction between fungal treatment and soil sterility regarding day 40 p.f.e. EPG (*F*_(1, 106)_ = 5.17, *P* < 0.0250) as the overall mean EPG in group SF hens was significantly higher than in groups SC (*P* < 0.0001) and NC hens (*P* = 0.0241) but comparable to group NF hens (*P* = 0.4040) (Fig. 5).

### *Ascaridia galli* IgY titres

**Fig. 6:**
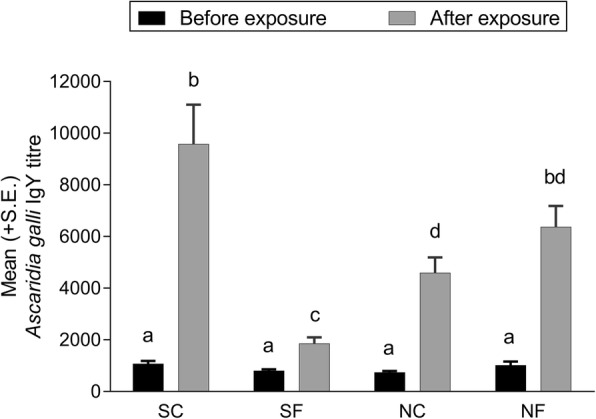
Mean (+ SE) *Ascaridia galli* IgY titre at one day before and 36 days after the first (of the total four) in-feed exposures to ascarid eggs embryonated in sterilised control soil (SC), sterilised soil with the fungus *Pochonia chlamydosporia* Biotype 10 (SF), non-sterilised control soil (NC) or non-sterilised soil with *P. chlamydosporia* (NF). Each bar represents the mean of 28–30 hens allocated into three replicate subgroups of 8 (one NC subgroup), 9 (two SC and two NF subgroups) or 10 hens. Different italicised letters above the bars indicate significant differences (*P* < 0.05, Tukey-Kramer’s adjustment for multiple comparisons) between the log-transformed titres

The overall group mean (+ SE) *A. galli* IgY titres in hens in the four groups on days -1 and 36 p.f.e. are shown in Fig. 6. All hens were seropositive at both time-points. The antibody titer was significantly affected by the interaction between sterility of soil, fungal treatment and sampling time (*F*_(4, 212)_ = 14.02, *P* < 0.0001). On day -1 p.f.e., the group mean ± SE titres ranged between 734 ± 62 and 1071 ± 115, with no significant differences between the groups (*P* > 0.7380 in all cases). By day 36 p.f.e., the IgY titre had increased significantly in all groups with an overall 8–11 fold increase in groups SC (*P* < 0.0001), NC (*P* < 0.0001) and NF (*P* < 0.0001), but only three fold increase in group SF hens (*P* < 0.0001). There were no significant correlations (*P* > 0.05) between IgY titre and individual worm burden (total ascarid, total *A. galli*) in all groups except SF where there were significant but weak correlations for the total ascarid (*r*_(30)_ = 0.38, *P* = 0.0362) and *A. galli* worm burden (*r*_(30)_ = 0.40, *P* = 0.0275).

## Discussion

The present study has for the first time shown that ascarid transmission to hens exposed to egg contaminated soil can be reduced after the soil has been treated with the fungus *P. chlamydosporia*, but only in sterilised soil. The reduced exposure resulted in a higher rate of development into adult worms and thus more patent infections compared to the more heavily infected control hens.

The fungus *P. chlamydosporia* Biotype 10 substantially reduced the egg recovery in the sterilised soil whereas in non-sterilised soil there was no additional effect when compared to the corresponding controls. This limited effect of *P. chlamydosporia* in the non-sterilised soil is in line with previous findings for egg-degrading fungi in general [38, 42, 52, 53]. The currently available literature indicates that native soil biota can reduce the establishment of a newly added fungus [54–58]. This is probably because the new fungus must compete for the soil resources or overcome antagonism by native established soil biota such as other fungi [59–61], bacteria [62–65], protozoa [66], free-living nematodes [67, 68], mites and dipteran larvae [69]. In future studies, application of fungi in nutrient-rich substrates (e.g. rice or barley kernels, decomposed resources etc.) could be explored as this may help increase fungal establishment in soil [55, 70, 71]. Ascarid eggs are sensitive to dessication [72] and after incubation, we found a slightly higher moisture loss in the sterilised soil compared to the non-sterilised soil. However, results indicate that this had no major impact as the moisture loss in the control and the fungus-treated sterilised soil was not significantly different and both the highest moisture loss and highest egg recovery from a single Petri dish was found in the sterilised soil.

The differences in soil egg numbers (i.e. exposure levels) after fungal treatment was reflected *in vivo* by parasite burden, establishment rate and population compostion within the host. The least exposed group had the lowest ascarid worm burdens but a higher parasite establishment rate compared to the three other groups that were more heavily exposed. Similar findings have been reported for an *A. galli* trickle infection in chickens [32], *H. gallinarum* single infection in red-necked pheasants [73] and *Oesophagostomum dentatum* single and trickle infections in pigs [74–76]. In contrast, Permin et al. [77] found no differences in *A. galli* burdens following a single dose of 100, 500 or 2500 eggs. This may be because they only quantified the luminal worms and many larvae in the two higher dose groups may potentially have been, at least temporarily, arrested in the intestinal mucosa [43, 44]. In the current study, we thus found an increased proportion of tissue phase *A. galli* presumably at the third larval stage (L3) [43, 44] in the three high exposure groups, and larger worms and patent infections primarily in the lowest exposure group. The absence of patent infections in most heavily infected hens supports that faecal egg counts can severely underestimate immature worm burdens and exposure levels [44, 74, 76].

The current results showed that low exposure may at least, in the short-term, lead to mature *A. galli* populations in contrast to predominantly immature infections at higher exposure levels. Reduced exposure and lower worm burdens are both desireable to lower the overall impact of ascarids on chicken health and productivity but seem to favour the establishment of patent infections. Density-dependent worm maturation was previously documented for *H. gallinarum* in chickens and ring-necked pheasants where heavily infected birds hosted significantly smaller female worms compared to lightly infected birds [73, 78]. It is unknown if, given time, at least some of our arrested larvae, presumably L3 in the intestinal tissue and L4 in the intestinal lumen [1, 43], would have reached maturity as our hens were only followed for 30 days after the last exposure. However, Ikeme [44] found the development of nearly all L3 to be arrested for up to 13 weeks post-last-exposure in birds that received a high infection dose. It is therefore very important to use sensitive recovery techniques to minimize the risk of overlooking high immature worm burdens. The precise mechanisms responsible for density-dependent effects are not fully understood. The combination of intraspecific competition among worms for limited resources (e.g. space, nutrients) in the host gut and the effect of host immune responses on parasite population seem important [79].

The above findings highlight the basic complication of any control strategy that cannot inactivate all parasite eggs in the environment. Initially there may be a lowered impact on the hosts present at the time due to lowered exposure, but if the result is associated with altered infection dynamics, and thus an earlier onset of patency, environmental recontamination might be higher than if there had been no intervention. This is further complicated as freshly deposited eggs take weeks to months to develop to infectivity depending on weather and season [30]. This goes to show that designing and implementing control strategies on a farm must take parasite biology and ecology into account to not only offer temporary relief, but also be effective long-term.

There is a close phylogenetic relationship between *A. galli* and *Heterakis* spp. [80] with corresponding production of cross-reacting antibodies [81]. However, the current contribution in the IgY titre due to *H. gallinarum* is expected to be neglible due to the much lower worm burdens compared to *A. galli*. The individual antibody levels appeared to increase with increasing exposure level and worm burden. However, individual antibody levels seemed uninvolved in any immune-related short-term regulation of *A. galli* populations. This is in agreement with previous findings of a very weak or a complete lack of correlation between systemic/egg-yolk IgY level and *A. galli*/*H. gallinarum* worm burden [3, 81]. A similar lack of association between porcine blood IgG level and worm burdens has been reported for *Ascaris suum* [82, 83] and *Trichuris suis* [84]. Furthermore, we also found that previous exposure did not protect against subsequent *A. galli* reinfection, which is in line with other studies [32, 85]. Others have reported increased mRNA expression of Th2 cytokines IL-4 and IL-13 in the intestinal tissues and spleen of *A. galli* infected hosts [3, 86, 87]. Both cytokines play a role in mediating protective immunity against several helminth parasites [88, 89] but it appears that *A. galli* may evade host immune responses to avoid expulsion as suggested for *O. dentatum* in pigs [90, 91]. This could be a reason why *A. galli* prevalence in laying hens kept in non-cage systems (barn, free-range and organic) seems to increase over time during an egg laying period of approximately one year [34].

To the best of our knowledge, there are no optimal/standardized protocols to establish patent *A. galli* infections in chickens. Experimental infection procedures vary greatly in relation to infection material (source, embryonation medium, temperature and duration of embryonation) and host factors (age, breed, etc.) [10, 32, 92–96]. This makes it extremely difficult to compare results between different studies. Many experiments performed earlier by our group could not establish patent *A. galli* infections when chickens were either infected with a single dose of 500 eggs [97, 98] or trickle infected twice weekly with 25–100 eggs per infection over a period of six weeks [32, 85]. The lowest infection dose used in the latter studies is very similar to the lowest exposure level of the current study. We have therefore made some modifications in the current protocol in relation to the previous failures. Hens were exposed to ascarid eggs at 20–22 weeks of age. This corresponded to the period when most hens started to lay eggs and it has been suggested that hens during this period are more susceptible and may have an increased establishment of *A. galli* due to hormonal changes in the birds [92]. Furthermore, we embryonated (i.e. incubated) ascarid eggs at 22 °C for only five weeks compared to the six week incubation protocol in the earlier studies. This was because chicken ascarid eggs develop fully within four weeks of incubation at 22 °C [29] and we thus provided only one additional week for the developed eggs to mature to infectivity.

## Conclusions

The Biotype 10 strain of *P. chlamydosporia* was only effective in inactivating ascarid eggs and thereby reducing the infection level of sterilised soil and thus total worm burdens of the exposed hens. The consequence was that the proportion of early mature/mature *A. galli* increased and faecal eggs counts were higher than in all the other groups where hens were exposed to a higher number of infective eggs in the soil. This underlines an inherent dilemma and complexity of ascarid control in that hosts may suffer less short-term, but current reduced exposure may lead to long-term higher environmental re-contamination if not all eggs can be eliminated. This needs to be considered in future biological and other control strategies in poultry.

## Abbreviations

EPG: eggs per gram of faeces
FEC: faecal egg counts
SE: standard error
